# Enhanced Thermal Conductivity and Dielectric Properties of Iron Oxide/Polyethylene Nanocomposites Induced by a Magnetic Field

**DOI:** 10.1038/s41598-017-03273-z

**Published:** 2017-06-08

**Authors:** Qingguo Chi, Tao Ma, Jiufeng Dong, Yang Cui, Yue Zhang, Changhai Zhang, Shichong Xu, Xuan Wang, Qingquan Lei

**Affiliations:** 1 Key Laboratory of Engineering Dielectrics and Its Application, Ministry of Education, Harbin University of Science and Technology, Harbin, 150080 P.R. China; 2 School of Applied Science, Harbin University of Science and Technology, Harbin, 150080 P.R. China; 3 State Key Laboratory of Electronic Thin Films and Integrated Devices, University of Electronic Science and Technology of China, Chengdu, 610054 P.R. China; 4 Key Laboratory of Functional Materials Physics and Chemistry of the Ministry of Education, Jilin Normal University, Siping, 136000 P.R. China

## Abstract

Iron Oxide (Fe_3_O_4_) nanoparticles were deposited on the surface of low density polyethylene (LDPE) particles by solvothermal method. A magnetic field was introduced to the preparation of Fe_3_O_4_/LDPE composites, and the influences of the magnetic field on thermal conductivity and dielectric properties of composites were investigated systematically. The Fe_3_O_4_/LDPE composites treated by a vertical direction magnetic field exhibited a high thermal conductivity and a large dielectric constant at low filler loading. The enhancement of thermal conductivity and dielectric constant is attributed to the formation of ﻿the conductive chains of Fe_3_O_4_ in LDPE matrix under the action of the magnetic field, which can effectively enhance the heat flux and interfacial polarization of the Fe_3_O_4_/LDPE composites. Moreover, the relatively low dielectric loss and low conductivity achieved are attributed to the low volume fraction of fillers and excellent compatibility between Fe_3_O_4_ and LDPE. Of particular note is the dielectric properties of Fe_3_O_4_/LDPE composites induced by the magnetic field also retain good stability across a wide temperature range, and this contributes to the stability and lifespan of polymer capacitors. All the above-mentioned properties along with the simplicity and scalability of the preparation for the polymer nanocomposites make them promising for the electronics industry.

## Introduction

Owing to the rapid developments in modern science and technology, the capacitors with remarkable performance were urgently required in the field of electronics and electrical systems. Nowadays, outstanding-performance polymers, which possess a high dielectric constant and flexibility, but low dielectric loss, have attracted great attention owing to their potential application in many cutting-edge industries, including microelectronics, aerospace, and aviation^1–3^. In general, the polymers possess excellent flexibility and high breakdown strength, but their applications were limited by their low dielectric constant^4, 5^. The organic-inorganic hybrid strategy, including ceramic/polymer and conductive fillers/polymer composites have been widely adopted^6–10^. Unfortunately, the ceramic/polymer composites have a limited dielectric constant and often require a large filler loading (>60 vol.%), which causes poor flexibility and uniformity of the materials. The conductive fillers/polymer composites can exhibit very high dielectric constant, but with a relatively high dielectric loss due to the insulator-conductor transition near the percolation threshold, which significantly restricts its practical application.

With the miniaturization of microelectronics and associated increase in power densities, the internal thermal stability of integrated microelectronic devices has become one of the critical factors in achieving advanced performance and extended lifespan^11–13^. It is crucial for the heat generated from electronics to be dissipated as quickly and effectively as possible, which can maintain the operating temperatures of the electronic equipment at a desired level, because the dielectric strength will decrease with increasing temperature owing to the poor thermal conduction of these dielectric materials. Notably, traditional high dielectric composites would lose their electromechanical stability and displayed a large variation in dielectric constant as well as dielectric loss at a broad temperature range, hindering their reliability and efficiency^14^.

Previous studies have shown that the polymeric composites, which were integrated with high thermal conductivity fillers, su﻿ch ﻿as metal (Cu)^15^, oxide(Al_2_O_3_)^16^, aluminum nitride (AlN)^17^, carbide(SiC)^18^﻿, and carbon nanotubes^19^, can endow themselves with superior properties. For example, Cu-filled low-density polyethylene (LDPE) composites were studied by Luyt *et al*.^15^ and they found that the thermal conductivity of the composites was 0.35 W m^−1^ K^−1^ when the volume fraction of the Cu particles was 7.0 vol.%. Fang *et al*.^18^ prepared different dimensional SiC particles to be filled into LDPE composites, and realized that the thermal conductivity of the composites was 0.37 W m^−1^ K^−1^ at 10 vol.% SiC content. High thermal conductivity of the composites usually requires a high volume fraction of fillers, and provision of a low dielectric constant, which is not suitable for use for microelectronics. Additionally, most research has just focused on one single side of thermal conductivity or dielectric property of nanocomposites at room temperature. Few in-depth explorations of the cooperative effect of a large thermal conductivity and a high dielectric constant for polymer materials under broader temperature conditions have been investigated until now, and their influential mechanism is still uncertain. Beyond that, how to improve the thermal conductivity and the dielectric performance of composites at low filler loading is one of the key issues.

The external electric and magnetic field could significantly influence the polymer’s molecular arrangement and the conductive particles’ distribution of the polymer composites, in the end the microstructure and macro-properties of the composites are influenced^20–22^. Hence, in this research, the Fe_3_O_4_-LDPE particles were prepared by solvothermal reaction and then the surface was modified by polydopamine (PDA). On this basis, we treated the Fe_3_O_4_/LDPE composites under a constant magnetic field for 30 min at 130 °C. Henceforth, the composites of Fe_3_O_4_/LDPE treated by the magnetic field can be abbreviated as M-Fe_3_O_4_/LDPE, here, “M” means that there has been magnet field treatment applied on the samples. The influence of the external magnetic field on the dielectric properties and thermal conductivity of Fe_3_O_4_/LDPE composites were studied intensively. It can be found that the orientation of the Fe_3_O_4_ nanoparticles was remote controlled by the external magnetic field because of its ultra-high magnetic response. Compared with the Fe_3_O_4_/LDPE composites, the M-Fe_3_O_4_/LDPE composites exhibit higher thermal conductivity and dielectric properties at 7.0 vol.% concentration, and the relevant mechanisms are discussed in detail.

## Results and Discussion

### Morphology of Fe_3_O_4_-LDPE particles and LDPE composites

**Figure 1:**
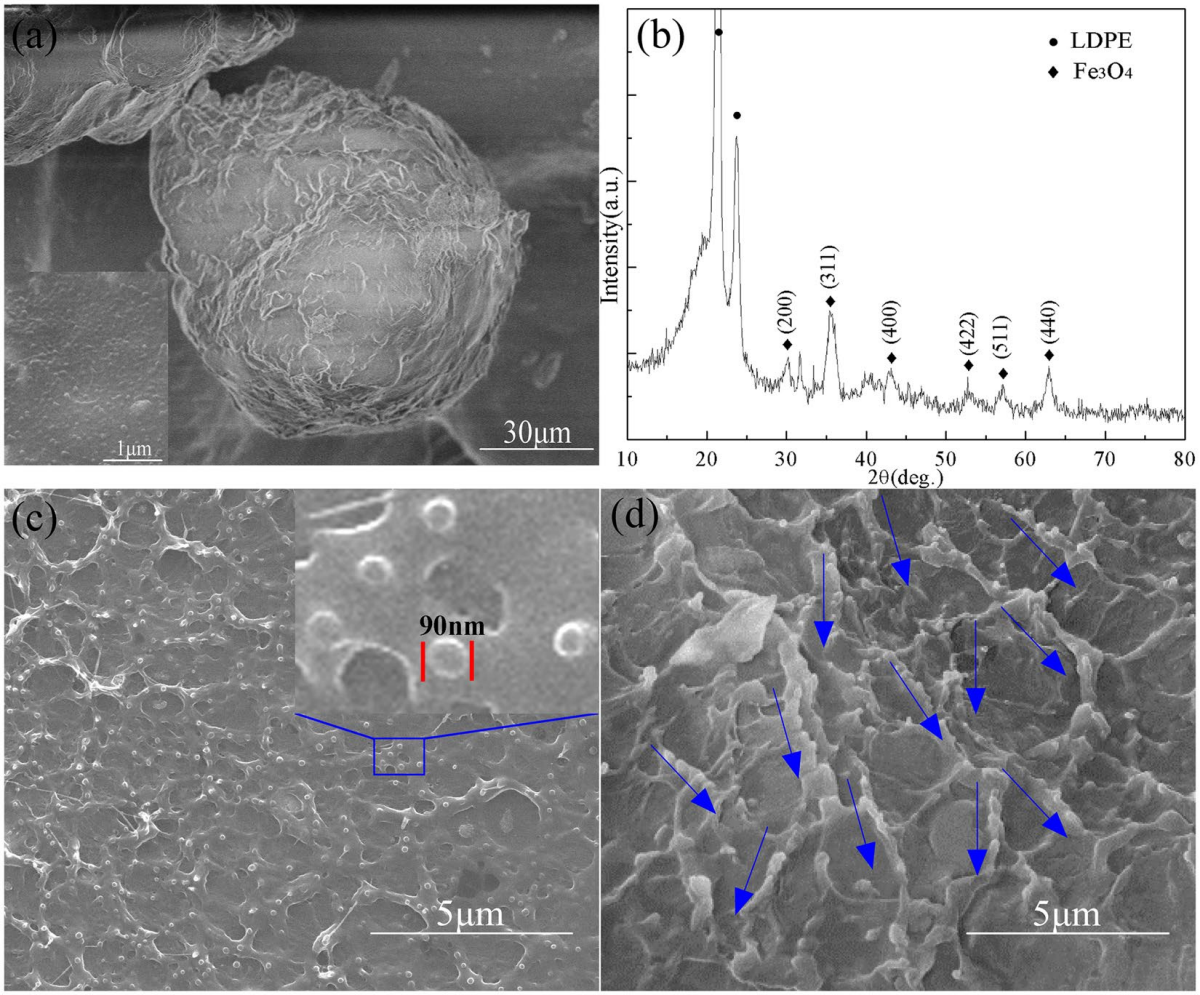
(**a**) SEM images of the Fe_3_O_4_-LDPE particles. The inset shows the partial SEM image of the Fe_3_O_4_-LDPE particles. (**b**) XRD pattern of the Fe_3_O_4_/LDPE composites at 7 vol.% concentration. (**c**) Cross-sectional SEM images of the Fe_3_O_4_/LDPE composites at 7 vol.% concentration. The inset shows the partial SEM image of the Fe_3_O_4_/LDPE composites. (**d**) Cross-sectional SEM images of the M-Fe_3_O_4_/LDPE composites at 7 vol.% concentration.

The morphology of the Fe_3_O_4_-LDPE particles is directly illustrated by scanning electron microscope (SEM) images, as shown in Fig. 1a. The LDPE particles have a non-spherical shape and with an average diameter of about 90 μm, and the Fe_3_O_4_ nanoparticles are uniformly deposited on LDPE particles. Figure 1b shows the X-ray diffraction (XRD) curves of the Fe_3_O_4_/LDPE composites with 7.0 vol.% Fe_3_O_4_ fillers. The characteristic diffraction peaks of Fe_3_O_4_ appear at 2θ = 30.18°, 35.56°, 43.24°, 53.54°, 57.10°, and 62.47°, corresponding to the diffraction peaks from (220), (311), (440), (422), (511), and (440), respectively. The XRD patterns of the composites clearly indicate that the Fe_3_O_4_ particles filled the polymer matrix.

The SEM image of the freeze-fractured cross-sections of the LDPE-based composite films containing 7 vol.% Fe_3_O_4_ nanoparticles is displayed in Fig. 1c. The Fe_3_O_4_ nanoparticles with an the average size of about 90 nm are embedded in the LDPE matrix without agglomeration, and there is no evidence of defects and voids in the Fe_3_O_4_/LDPE composites, which is due to the solvothermal reaction process of the Fe_3_O_4_-LDPE particles lowering the probability of the agglomeration of the Fe_3_O_4_ nanoparticles. The surfactant treatment of PDA greatly improved the compatibility of the Fe_3_O_4_ nanoparticles and LDPE matrix^23^. Figure 1d shows the SEM morphology of the fractured cross-surface of M-Fe_3_O_4_/LDPE composite at 7 vol.% concentration. It is clearly found that some Fe_3_O_4_ particles come into contact with each other and become short chains along the magnetic direction. That is, the distribution of the Fe_3_O_4_ particles in the LDPE matrix is changed and the chains of the Fe_3_O_4_ particles are formed under the action of the magnetic field.

### Thermal conductivity of Fe_3_O_4_/LDPE composites and M-Fe_3_O_4_/LDPE composites

**Figure 2:**
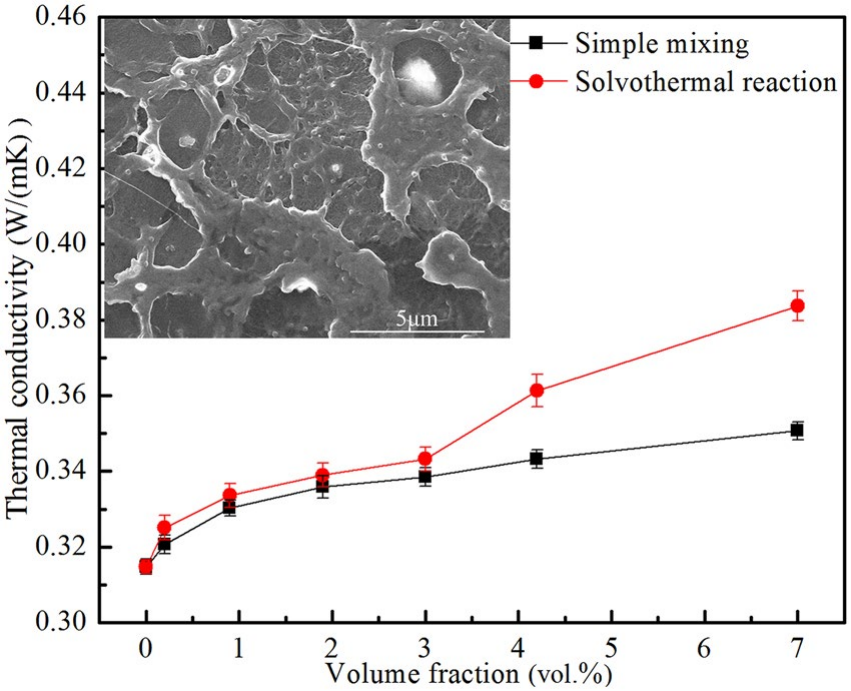
Comparison of thermal conductivity of Fe_3_O_4_/LDPE composites prepared by simple mixing method and solvothermal reaction. The inset shows the cross-sectional SEM images of the Fe_3_O_4_/LDPE composites at 7 vol.% concentration prepared by simple mixing method.

In this research, the Fe_3_O_4_-LDPE particles were prepared via a solvothermal reaction before preparing the Fe_3_O_4_/LDPE composites. To highlight the advantages of this method, the comparison of the thermal conductivity of the Fe_3_O_4_/LDPE composites prepared by a simple mixing method and solvothermal reaction is shown in Fig. 2. Compared with the simple mixing method, the Fe_3_O_4_/LDPE composites prepared by solvothermal reaction show a higher thermal conductivity at the same filler content. For instance, the maximum thermal conductivity of the Fe_3_O_4_/LDPE composites via the solvothermal reaction is 0.384 W m^−1^ K^−1^ at 7.0 vol.% concentration, higher than that of Fe_3_O_4_/LDPE composites prepared by a simple mixing method (0.350 W m^−1^ K^−1^). As shown in the inset of Fig. 2, the voiding occurs due to the agglomeration and the weak compatibility of the Fe_3_O_4_ nanoparticles and LDPE matrix prepared by the simple mixing method, which leads to poor thermal conductivity of the Fe_3_O_4_/LDPE composites. This also indicated that the solvothermal treatment could effectively improve the dispersion of the Fe_3_O_4_ nanoparticles in the LDPE matrix.

**Figure 3:**
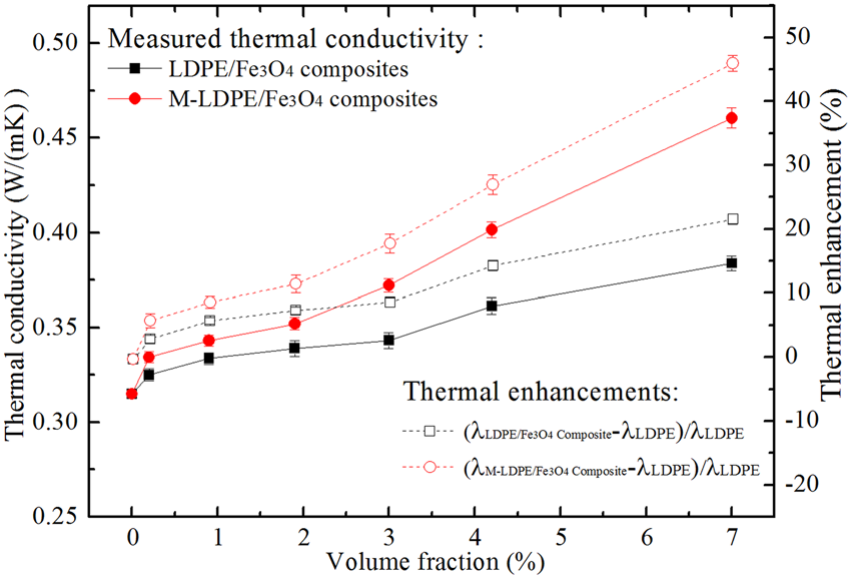
Thermal conductivity of the Fe_3_O_4_/LDPE and M-Fe_3_O_4_/LDPE composites corresponding to thermal enhancements.

The curves of thermal conductivity of the composites versus filler content are displayed in Fig. 3. All of the composites show an enhancement in thermal conductivity with increasing Fe_3_O_4_ concentration. Moreover, a non-linear increase in thermal conductivity is also observed. The higher thermal conductivity of the composites should result from the higher intrinsic thermal conductivity of the Fe_3_O_4_ particles in comparison with the LDPE matrix^24, 25^. Importantly, the thermal conductivity of the M-Fe_3_O_4_/LDPE composites are much higher than that of the Fe_3_O_4_/LDPE composites at the same loading. For instance, the thermal conductivity of the M-Fe_3_O_4_/LDPE composites is 0.461 W m^−1^ K^−1^ at 7.0 vol.% concentration, higher than that of the Fe_3_O_4_/LDPE composites (0.384 W m^−1^ K^−1^), and this value is superior to that of many previous reports^15, 18, 20, 26–28^. As shown in Table 1, the thermal conductivity of our composites is larger than that of the high-density polyethylene/fly-ash (HDPE/FA) composites containing 10 vol.% FA (≈0.41 W m^−1^ K^−1^)^27^. It should be pointed out that the filler content of the M-Fe_3_O_4_/LDPE composites is smaller than that of other literature materials, with better flexibility.

**Table 1:** Comparison of the thermal conductivity of our composites and reported literature materials.

Composites	*λ*(w/mk)	*f* (vol.%)	Ref.
Fe_3_O_4_/LDPE	0.384	7	Our work
M-Fe_3_O_4_/LDPE	0.461	7	Our work
LDPE-Cu	0.35	7	[15]
SiC (21 μm)/LLDPE	0.52	≈10.77	[18]
Rmh-BN-Silicone	0.349	7.5	[20]
AlN (A-100)/HDPE	0.46	10	[26]
HDPE/FA	≈0.41	10	[27]
AIN/Polyimide	0.75	32	[28]

Figure 3 also presents the thermal enhancements of the Fe_3_O_4_/LDPE and M-Fe_3_O_4_/LDPE composites with pure LDPE, respectively. Herein, the thermal enhancement is defined as the percentage of thermal conductivity improvement by Fe_3_O_4_^20, 29^. It can be found that the thermal enhancement of the Fe_3_O_4_/LDPE and M-Fe_3_O_4_/LDPE composites increases initially with the filler content, reaching 21.88% and 46.27% at 7.0 vol.% concentration, respectively. The M-Fe_3_O_4_/LDPE composites show a better heat conduction effect than the Fe_3_O_4_/LDPE composites, owing to the formation of efficient thermal pathways made by the Fe_3_O_4_ oriented parallel to the heat flux under the action of the magnetic field (see Fig. 1d).

To further explore the thermal conductivity behavior of the Fe_3_O_4_/LDPE and M-Fe_3_O_4_/LDPE composites, Maxwell-Eucken’s model has been proposed and the comparisons between the experimental and theoretical values were made^30^. Maxwell-Eucken assumes that filler particles are homogeneously distributed in the polymer matrix, non-interacting, and roughly spherical. Fewer filler particles would be covered by the polymer and dispersed in the form of isolated islands in the matrix:

1 $${\lambda }_{c}=\frac{2{\lambda }_{p}+{\lambda }_{f}+2{V}_{f}({\lambda }_{f}-{\lambda }_{p})}{2{\lambda }_{p}+{\lambda }_{f}-2{V}_{f}({\lambda }_{f}-{\lambda }_{p})}{\lambda }_{p}$$

where *V*_*f*_ is the volume fraction of the filler, and *λ*_*c*_, *λ*_*f*_, and *λ*_*p*_ is the thermal conductivity of composites, filler, and matrix, respectively^31, 32^. In this paper, the value of *λ*_*f*_ and *λ*_*p*_ used 6.032 W m^−1^ K^−1^ and 0.315 W m^−1^ K^−1^, respectively.

**Figure 4:**
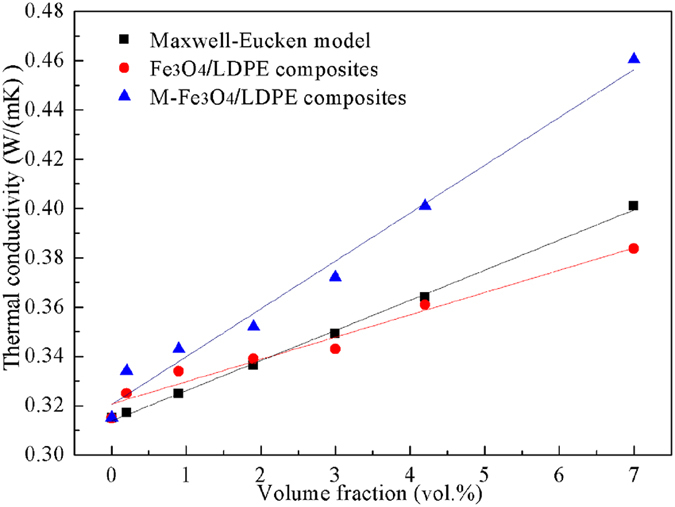
Thermal conductivity of the Fe_3_O_4_/LDPE and M-Fe_3_O_4_/LDPE composites based on experimental data and Maxwell-Eucken’s model.

Figure 4 gives the comparison between the experimental data and the thermal conductivity predicted by the Maxwell-Eucken’s model of the composites. For the Fe_3_O_4_/LDPE composites, Fe_3_O_4_ nanoparticles are dispersed randomly in the Fe_3_O_4_/LDPE composites (shown in Fig. 1c), and the theoretical values match well with the experimental data. However, it is interesting to note that the theoretical values of the M-Fe_3_O_4_/LDPE composites are lower than those observed in the experiments. This deviation could be attributed to the easier formation of the thermal conductive net-chain by the interaction of Fe_3_O_4_ in the LDPE matrix under the action of the magnetic field.

Owing to the Maxwell-Eucken model not matching well with the M-Fe_3_O_4_/LDPE composites, two heat conduction models (parallel and vertical models) were brought forward to judge the heat flow direction in the polymer composites, based on the formation of the thermal conductive net-chain and mutual interaction of particles^33, 34^. Considering the crystallinity of the filler and polymer, Agari’s model was created based on a hypothesis of homogeneous dispersion of particles in the polymer:

2 $$\mathrm{log}\,\lambda =V{C}_{2}\,\mathrm{log}\,{\lambda }_{f}+(1-V)\mathrm{log}\,({\lambda }_{p}{C}_{1})$$

where *C*_1_ is the constant, which is related to the crystallinity and crystalline dimension of a polymer, and *C*_2_ is the free factor, which indicates the ability of forming a heat conductive net-chain for fillers. *C*_2_ would significant change due to the increase of the particle filling; therefore, Agari’s model should be modified and verified as the following^32, 35^:

3 $$\mathrm{log}\,\lambda ={C}_{f}V\,\mathrm{log}\,[{\lambda }_{f}/({C}_{p}{\lambda }_{p})]+\,\mathrm{log}\,({C}_{p}{\lambda }_{p})=AV+B$$

where *C*_*p*_ is the formation of the thermal conductive chain free particles, and *C*_*f*_ is the reflecting particle formation of the thermal conductivity of difficulty.

Table 2 shows the *C*_*p*_ and *C*_*f*_ for the above two composites. It can be found that the *C*_*p*_ of the M-Fe_3_O_4_/LDPE composites did not change significantly compared with the Fe_3_O_4_/LDPE composites. However, the *C*_*f*_ of the M-Fe_3_O_4_/LDPE composites was greater than that of the Fe_3_O_4_/LDPE composites. The factor *C*_*f*_ related to the ease in forming conductive chains of the filler, that is, the magnetic field shows a strong effect on *C*_*f*_. This also indicates that the external magnetic field could effectively enhance the formation of thermal conductive paths in the LDPE matrix, which is consistent with the results shown in Fig. 1d. In addition, R^2^ stands for the fitting degree between the theoretical data and the experimental data. The R^2^ value of the M-Fe_3_O_4_/LDPE composites is 0.98522, higher than that of the Fe_3_O_4_/LDPE composites (0.96233), showing that the experimental results for the M-Fe_3_O_4_/LDPE composites had a better fitting effect than the Fe_3_O_4_/LDPE composites. Besides, as shown in Fig. 1d, some changes have taken place in the structure of the LDPE matrix. This phenomenon also indicated that the external magnetic field has some influence on the microstructure of polymer matrix, the similar result has been reported in numerous previous studies^36–38^. It may be one of the reasons for the thermal conductivity enhancement of M-Fe_3_O_4_/LDPE composites.

**Table 2:** Values of *C*_*p*_ and *C*_*f*_ for the Fe_3_O_4_/LDPE and M-Fe_3_O_4_/LDPE composites.

Composites	A	B	*C*_*p*_	*C*_*f*_	R^2^
Fe_3_O_4_/LDPE	1.11624	−0.55891	1.02028	0.87656	0.96233
M-Fe_3_O_4_/LDPE	2.19564	−0.49094	1.02505	1.72694	0.98522

### Dielectric properties of Fe_3_O_4_/LDPE composites and M-Fe_3_O_4_/LDPE composites

**Figure 5:**
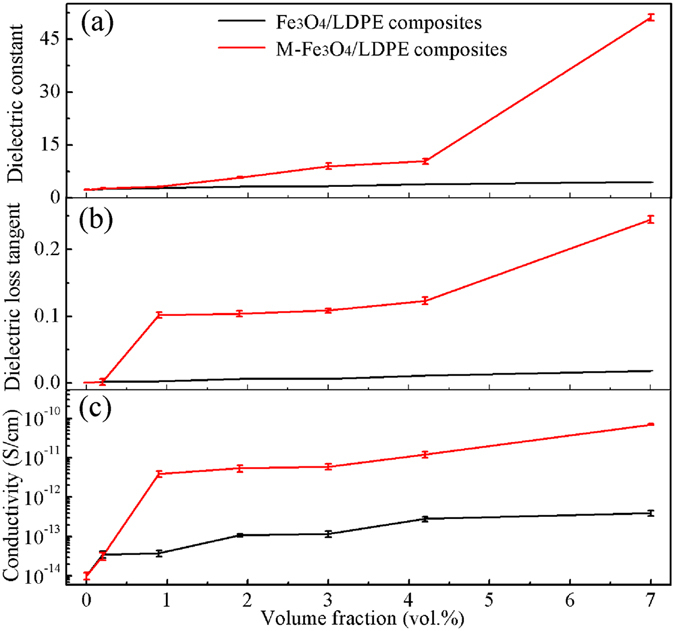
(**a**) Dielectric constant (**b**) dielectric loss tangent and (**c**) conductivity of the Fe_3_O_4_/LDPE and M-Fe_3_O_4_/LDPE composites at different volume fraction of Fe_3_O_4_ filler at 10 Hz.

The thermal conductivity of the Fe_3_O_4_/LDPE composites is enhanced by the formation of conductive chains of the Fe_3_O_4_ filler under the action of the magnetic field. Moreover, the distribution of the Fe_3_O_4_ nanoparticles also affects the dielectric properties of the corresponding materials. Figure 5 shows the denpendence of volume fraction and dielectric properties of the Fe_3_O_4_/LDPE and M-Fe_3_O_4_/LDPE composites at 10 Hz and room temperature. It can be found that the dielectric constant of the composites increases with gradually increasing filler content. The maximum dielectric constant of the Fe_3_O_4_/LDPE composites is 4.45 when the content of Fe_3_O_4_ is 7.0 vol.%. In comparison with pure LDPE (*ε* = 2.3), the dielectric constant of the composite is nearly 2 times higher. This demonstrates that incorporating conducting fillers into the polymer matrix results in an increase in dielectric constant. Moreover, the dielectric constant of the M-Fe_3_O_4_/LDPE is higher than the Fe_3_O_4_/LDPE composites at the same concentration. In particular, the dielectric constant of the M-Fe_3_O_4_/LDPE composites reaches 51 at 7.0 vol.% concentration, which is 11.6 times higher than that of the Fe_3_O_4_/LDPE composites. Fe_3_O_4_ nanoparticles are able to move easily and touch together to form clusters in a melting state of the polymer matrix (at 130 °C), and these clusters would array in parallel along the direction of the magnetic field^21, 22^. The oriented distribution of the Fe_3_O_4_ clusters is beneficial for the formation of a conductive network in this direction, which can be regarded as a great amount of parallel-connected micro-capacitors, resulting in a greatly enhanced dielectric constant^39, 40^. It should be noted that the dielectric properties in our present study is even higher than those of many previous reports^21, 23, 41–43^. For example, as shown in Table 3, the obtained dielectric constant is higher than that of BZT-BCT/PVDF composites containing 24 vol.% BZT-BCT (*ɛ* = 37.2 at 10 Hz)^41^. Moreover, the amount of filler in the M-Fe_3_O_4_/LDPE composites is smaller than that in other materials described in the literature, and displayed better flexibility. For embedded capacitor applications, the dielectric loss is an essential parameter. The dielectric loss measured at a certain frequency includes polarization loss and conduction loss^44^. The dielectric loss tangent for the M-Fe_3_O_4_/LDPE composites remain below 0.25 at 10 Hz, and the conductivity of the M-Fe_3_O_4_/LDPE composites is also kept at a low value (7 × 10^−11^ S/cm). These attributed to the improvement of the interfacial adhesion and compatibility between the filler and the matrix by surface treatment of the PDA, no complete conducting path was formed in the composites (shown in Fig. 1d), resulting in a low dielectric loss and low conductivity of the composites within the acceptable ranges^45^. For example, the achieved dielectric loss and conductivity were found to be significant smaller than that of PEG-Fe_3_O_4_/PVDF composites containing 7.5 vol.% PEG-Fe_3_O_4_ fillers^43^.

**Table 3:** Comparison of the dielectric properties of our composites and reported literature materials.

Composites	*ε*	tan *δ*	*σ* (S/cm)	*f* (vol.%)	Ref.
Fe_3_O_4_/LDPE	4.45	0.0188	3.96 × 10^−13^	7	Our work
M-Fe_3_O_4_/LDPE	51	0.25	7 × 10^−11^	7	Our work
PVDF/Ni	45	0.2	—	5	[21]
P(VDF-HFP)/BT-PDA-Ag	≈44	0.213	—	20	[23]
BZT-BCT/PVDF	37.2	0.08	1.5 × 10^−9^	24	[41]
Fe_3_O_4_/SiO_2_/BECy	9.5	0.09	—	20	[42]
PEG-Fe_3_O_4_/PVDF	≈80	≈1.25	≈7.0 × 10^−9^	7.5	[43]

In addition to the movement of particles under the magnetic field, the dielectric relaxation is also likely to affect the dielectric properties. To clarify the mechanism of the dielectric behaviors of the Fe_3_O_4_/LDPE and M-Fe_3_O_4_/LDPE composites, an electric modulus formalism based analysis of the dielectric relaxation in the composites has been proposed. Complex dielectric modulus formalism M* is demonstrated as follows:^46, 47^

4 $${M}^{\ast }=\frac{1}{{\varepsilon }^{\ast }}=\frac{1}{\varepsilon ^{\prime} -j\varepsilon ^{\prime\prime} }=\frac{\varepsilon ^{\prime} }{{\varepsilon ^{\prime} }^{2}+{\varepsilon ^{\prime\prime} }^{2}}+j\frac{\varepsilon ^{\prime\prime} }{{\varepsilon ^{\prime} }^{2}+{\varepsilon ^{\prime\prime} }^{2}}=M^{\prime} +jM^{\prime\prime} $$

where *M*′ is the real and *M*″ the imaginary part of the electric modulus, respectively. The imaginary part *M*″ of the electric modulus takes the form of loss curves, allowing us to interpret the relaxation phenomena.

**Figure 6:**
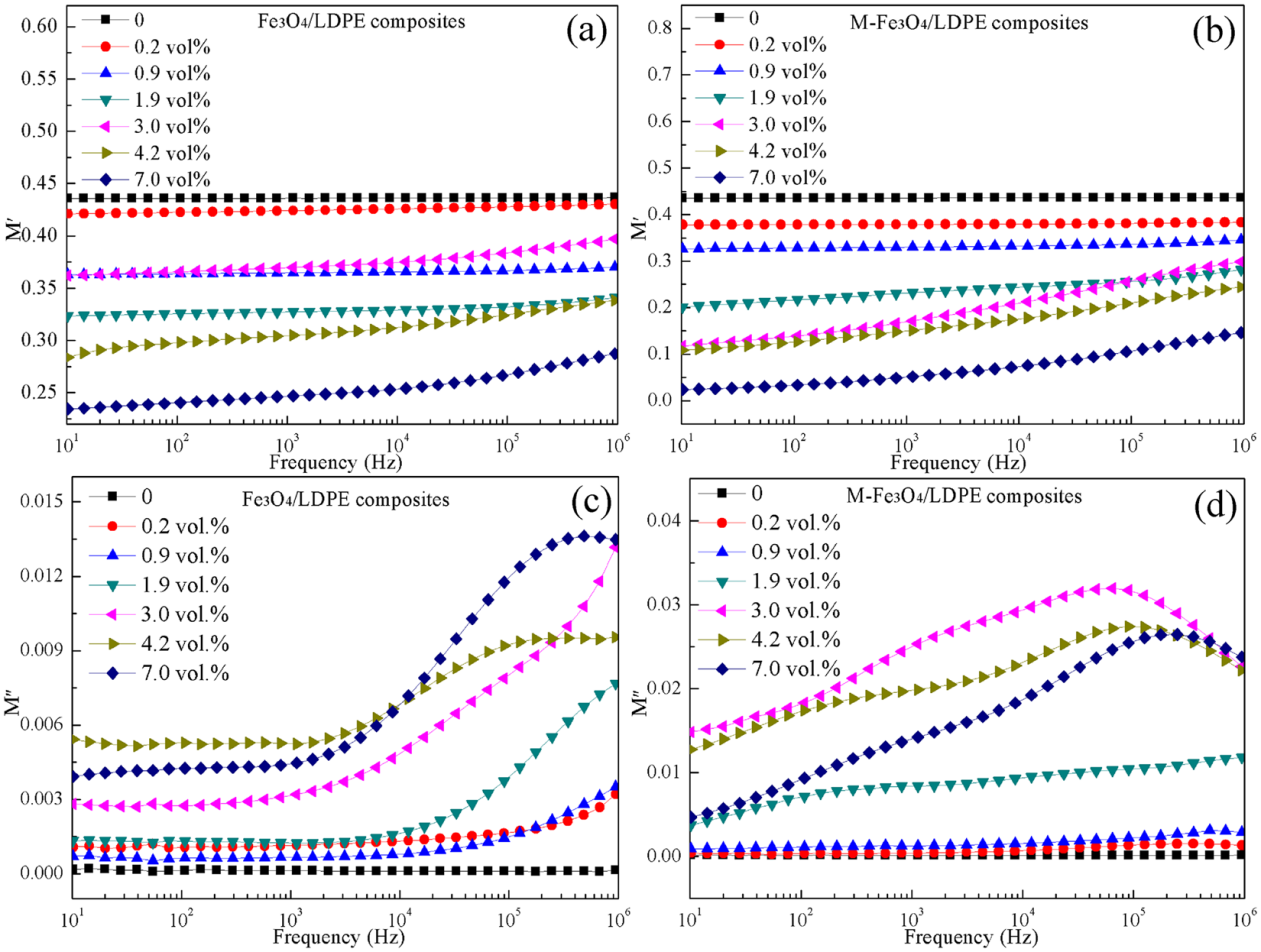
Frequency dependence of the (**a**,**b**) real parts and (**c**,**d**) imaginary parts of the complex electric modulus for the Fe_3_O_4_/LDPE and M-Fe_3_O_4_/LDPE composites.

The frequency dependence of *M*′ and *M*″ for the Fe_3_O_4_/LDPE and M-Fe_3_O_4_/LDPE composites is shown in Fig. 6. The change regularity of the Fe_3_O_4_/LDPE composites is insignificant with the increasing of Fe_3_O_4_ content (Fig. 6a). However, for the M-Fe_3_O_4_/LDPE composites, it can be seen that *M*′ decreases with the increasing of Fe_3_O_4_ content (Fig. 6b), and increases with frequency; this behavior is similar to other polymer composites containing conducting fillers^48, 49^. As shown in Fig. 6c and d, compared with the Fe_3_O_4_/LDPE composites, obvious interfacial polarization relaxation peaks occur for the M-Fe_3_O_4_/LDPE composites when the Fe_3_O_4_ content is higher than 1.9 vol.%. Moreover, the relaxation strength of the composites decreases with the increase of Fe_3_O_4_ loading, and the relaxation peak moved toward higher frequency as the Fe_3_O_4_ content increased. The inter-particle distance would decrease as the volume fraction of the Fe_3_O_4_ increased, and as a result, the probability of Fe_3_O_4_ nanoparticles coming into contact increased because of the external magnetic field. As shown in Fig. 1d, some Fe_3_O_4_ particles come into contact with each other and become short chains along the direction of the magnetic field, which leads to a higher possibility for the charge carriers to accumulate on the interface between Fe_3_O_4_ and LDPE, the polarization and dielectric response are greatly enhanced under the electric field^22^. Therefore, a high dielectric constant of M-Fe_3_O_4_/LDPE composites is achieved at a low volume fraction.

**Figure 7:**
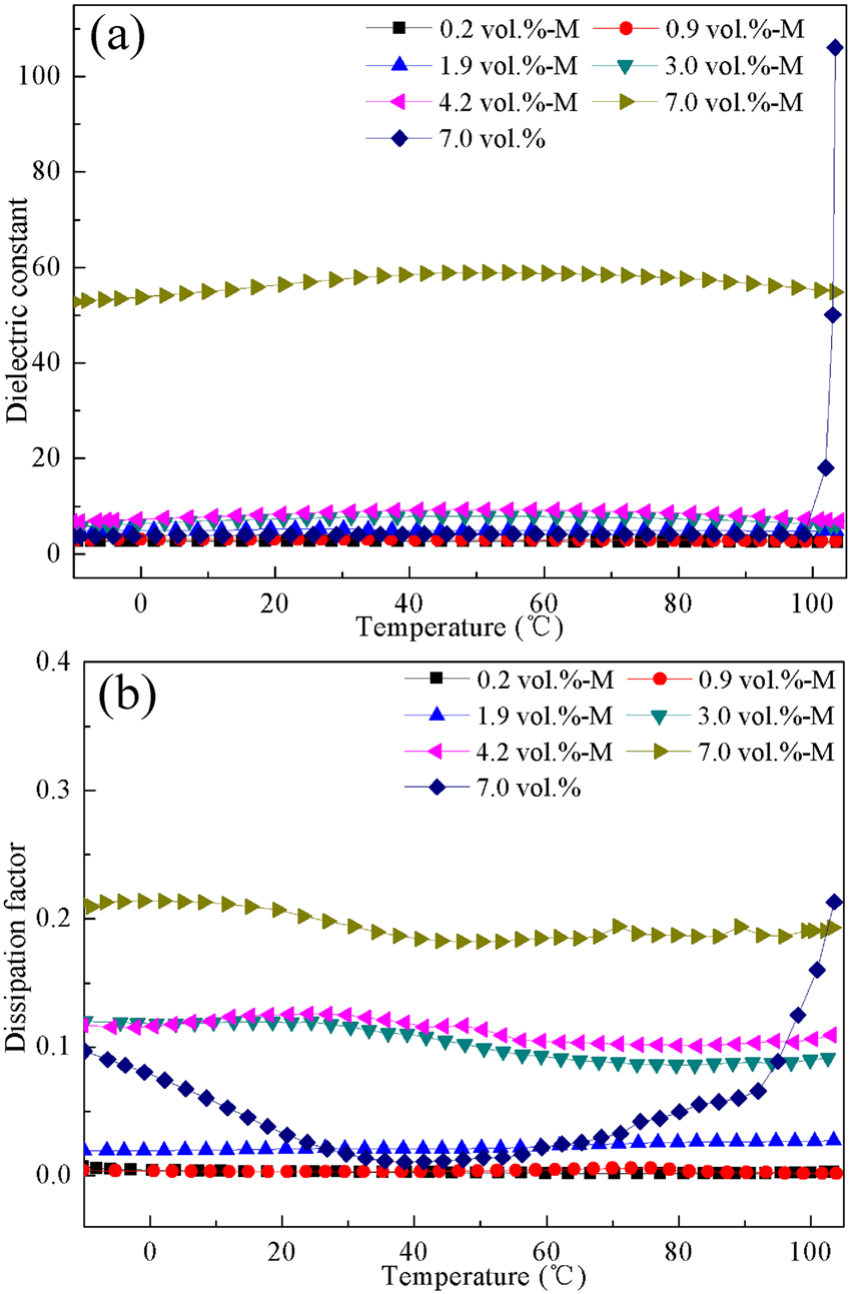
Temperature dependence of (**a**) dielectric constant and (**b**) dissipation factor of the Fe_3_O_4_/LDPE and M-Fe_3_O_4_/LDPE composites at different volume fraction of Fe_3_O_4_ filler at 10 Hz.

For polymer capacitors, it is essential that the dielectric properties of the composites retain excellent stability across a wide temperature range^11, 14^. In this research, the temperature dependence of the dielectric constant and dissipation factor of the Fe_3_O_4_/LDPE and M-Fe_3_O_4_/LDPE composites at 10 Hz are given in Fig. 7. It can be found that a minor variation in dielectric constant of the M-Fe_3_O_4_/LDPE composites occurs with the increasing of temperature. The M-Fe_3_O_4_/LDPE composites at 7.0 vol.% Fe_3_O_4_ concentration display the largest dielectric constant (>51) across the whole temperature ranges. However, for the Fe_3_O_4_/LDPE composites, an obvious increasing trend occurred when the temperature was over 100 °C. Concurrently, the dissipation factor of the M-Fe_3_O_4_/LDPE composites at 10 Hz also remained at a slightly low value and maintained good stability across the whole temperature range, but an appreciable change has been observed in the Fe_3_O_4_/LDPE composites at 7.0 vol.% concentration. It is also evident that, of the dielectric assessed, the M-Fe_3_O_4_/LDPE composites offer a stable dielectric constant and dissipation factor at high temperature. This is attributed to the fact that the higher thermal conductivity of the M-Fe_3_O_4_/LDPE composites can effectively depress the dielectric loss and conductivity. It also indicates that the magnetic field could largely enhance the dielectric-temperature stability of the Fe_3_O_4_/LDPE composites across broad temperature range. There is an undoubtable benefit to the use of Fe_3_O_4_/LDPE composites across a wider temperature range in electronics.

In our present study, Fe_3_O_4_ nanoparticles adopted a directional arrangement along the direction of the magnetic field, and formed a conductive network in the LDPE matrix under the action of a magnetic field, resulting in greatly enhanced thermal conductivity. Moreover, the interfacial polarization of the Fe_3_O_4_/LDPE composites is effectively enhanced under magnetic field treatment, and the maximum dielectric constant of the composites reaches 51 at 7.0 vol.% concentration. Additionally, the low volume fraction of the fillers and the excellent compatibility of the Fe_3_O_4_ nanoparticles and LDPE matrix, resulted in a relatively low dielectric loss (0.25) and a low conductivity (7 × 10^−11^ S/cm). Moreover, the dielectric properties of the M-Fe_3_O_4_/LDPE composites retain good stability across a wide temperature range (−10 °C~105 °C), resulting from the high thermal conductivity of the Fe_3_O_4_/LDPE composites induced by the magnetic field. This good heat dissipation capability prolonged the lifespan of the polymer dielectrics at a higher operating temperature.

## Conclusion

In summary, Fe_3_O_4_-deposited LDPE hybrid particles were prepared by a solvothermal reaction, and the corresponding Fe_3_O_4_/LDPE composites were also prepared. SEM images show that Fe_3_O_4_ nanoparticles were embedded in the LDPE matrix without agglomeration and defects. The magnetic field enhanced the probability of forming Fe_3_O_4_ conductive chains, which can effectively enhance the heat flux and interfacial polarization of Fe_3_O_4_/LDPE composites. The Fe_3_O_4_/LDPE composites induced by the magnetic field exhibited higher thermal conductivity and a higher dielectric constant in comparison with the Fe_3_O_4_/LDPE composites at the same filler content. Moreover, the relatively low dielectric loss and low conductivity are attributed to the low filler content and the improvement compatibility of the Fe_3_O_4_ nanoparticles and LDPE matrix by the surfactant treatment of the PDA. The low filler content leads to good mechanical properties and material processibility. Additionally, the dielectric properties of the M-Fe_3_O_4_/LDPE composites also retain good stability across a wide temperature range, due to the high thermal conductivity of the Fe_3_O_4_/LDPE composites induced by the magnetic field. This work establishes a facile, yet efficient approach to synthesize polymer materials with high thermal conductivity and high dielectric properties in order to make them suitable candidates for use in the electronics industry.

## Materials and Methods

### Preparation of Fe_3_O_4_ nanoparticles

The typical procedures for the preparation of the super-paramagnetic Fe_3_O_4_ nanoparticles were carried out as follows. Firstly, 1.442 g of FeSO_4_ • 7H_2_O and 2.472 g of FeCl_3_ • 6H_2_O were dissolved in 50 mL distilled water, respectively, then mixed together according to the mole ratio (Fe^3+^:Fe^2+^  = 2:1.134) to inhibit the oxidation of Fe^2+^. The iron solution and NaOH alkaline solution were slowly added to 100 mL distilled water in a 500 mL flask under vigorous stirring at 40 °C for 30 min, the pH of aqueous solution in the whole process was fixed at 12–13. After standing for about 1 h, the precipitate was washed with distilled water and ethanol until the pH reached neutral. The Fe_3_O_4_ nanoparticles were obtained and dried under vacuum at 60 °C for 12 h.

### Preparation of Fe_3_O_4_-LDPE particles

The typical procedures for the preparation of the super-paramagnetic Fe_3_O_4_ deposited on the LDPE particles (Fe_3_O_4_-LDPE) were carried out as follows. Firstly, the desired amount of LDPE particles was poured into 175 ml ethanol, followed by the addition of Fe_3_O_4_ under vigorous stirring at room temperature for 15 min. Then, the obtained mixture was transferred into a 200 ml Teflon-lined stainless steel autoclave. The solvothermal reaction was kept at 120 °C for 4 h, then the precipitate was collected, and washed with distilled water and ethanol until the pH around 7. Finally, the Fe_3_O_4_-LDPE particles were obtained and dried under vacuum at 50 °C for 12 h. The volume fractions of Fe_3_O_4_ in the Fe_3_O_4_-LDPE particles were 0.2 vol.%, 0.9 vol.%,1.9 vol.%, 3.0 vol.%, 4.2 vol.%, and 7.0 vol.%, respectively.

### Preparation of Fe_3_O_4_/LDPE composites

**Figure 8:**
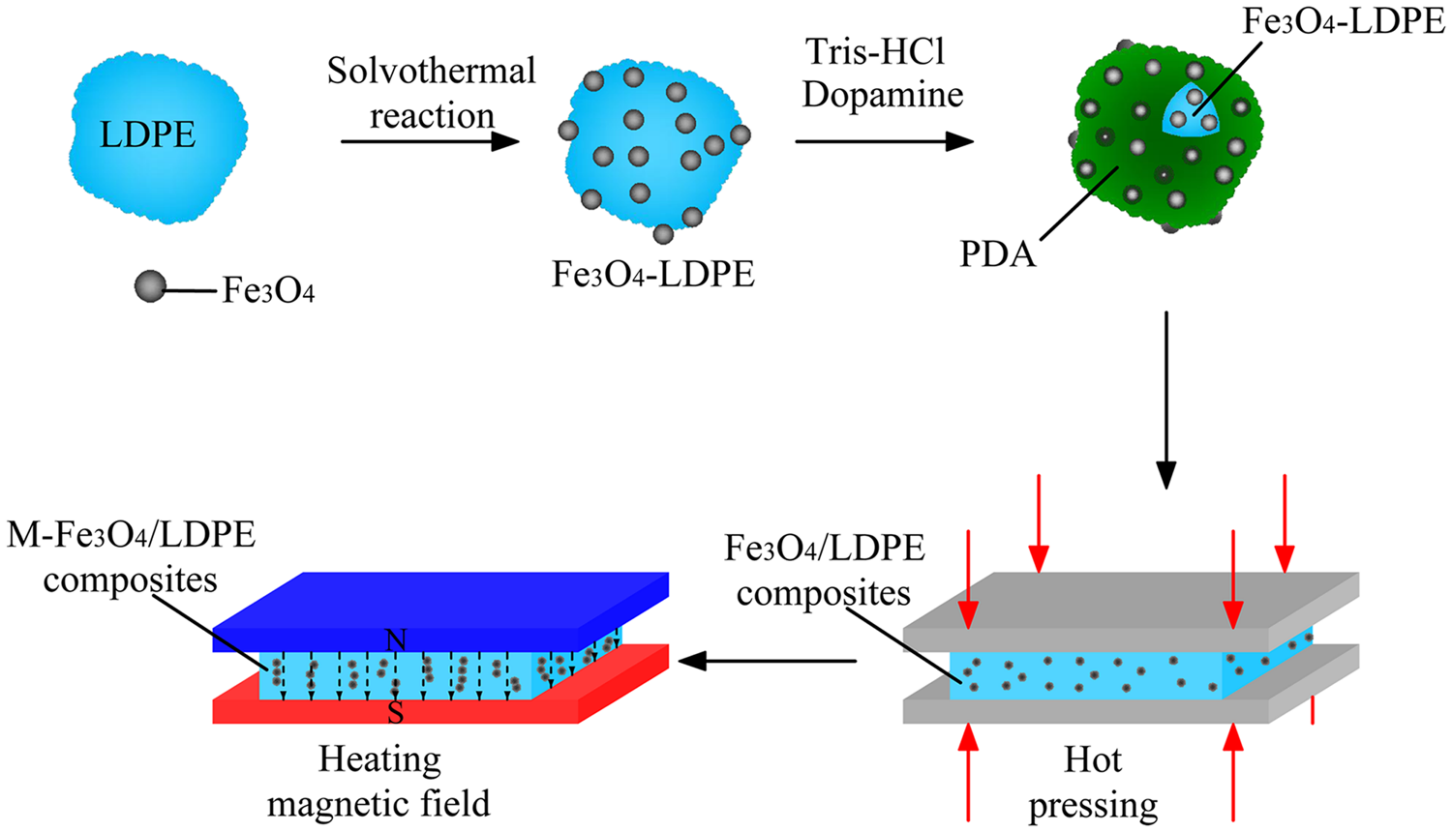
Schematic illustration of the preparation of the Fe_3_O_4_/LDPE and M-Fe_3_O_4_/LDPE composites.

Before preparing the composites, the 0.6 g Fe_3_O_4_-LDPE particles were dispersed in a 200 mL Tris-HCl aqueous solution (10 × 10^−3^ M, pH = 8.5) followed by sonication for 1 h; then 0.2 g DA-HCl was added and sonicated for another 10 min. The mixture was then stirred vigorously at room temperature for 4 h, and the excess supernatant was removed after sedimentation for about 1 h. The Fe_3_O_4_-LDPE particles were obtained and dried under vacuum at 50 °C for 12 h. The Fe_3_O_4_/LDPE composites were obtained by a torque rheometer for 30 min at 130 °C and then molded by hot pressing at approximately 130 °C and 15 MPa for 20 min. The M-Fe_3_O_4_/LDPE composites were obtained by treating at 130 °C under a vertical direction magnetic field with a magnetic induction density of 1.0 T for 30 min. The preparation process of the Fe_3_O_4_/LDPE and M-Fe_3_O_4_/LDPE composites is shown in Fig. 8.

### Characterization

The morphology of the Fe_3_O_4_-LDPE particles and the microstructure of the LDPE composites were observed using SEM (Quanta 200 FEI). The phase compositions of the Fe_3_O_4_/LDPE composites were analyzed using XRD (Empyrean), using Cu Kα radiation at 40 kV and 40 mA. The thermal diffusivity (*α*) and specific heat (*C*) were measured on the disk samples with an LFA447 light flash system (NETZSCH, Selb, Germany) at 25 °C. The thermal conductivity was calculated by *λ* = *αCρ*, in which *ρ* is the density of the Fe_3_O_4_/LDPE composite. Prior to performing dielectric measurements, a layer of Al paste (diameter 25 mm) was evaporated on both surfaces to serve as electrodes. Dielectric measurements were employed across a frequency range from 10 to 10^6^ Hz and a temperature range of −10 °C to 105 °C using a broad frequency dielectric spectrometer (Novocontrol Alpha-A).
